# Decoding the identity of rare tumors

**DOI:** 10.7554/eLife.102833

**Published:** 2024-10-07

**Authors:** Qingchen Yuan, Prabhjot Kaur, Olga A Guryanova

**Affiliations:** 1 Department of Pharmacology and Therapeutics, University of Florida College of Medicine Gainesville United States

**Keywords:** solitary fibrous tumors, neuroendocrine tumors, gene fusion, targeted medicine, Human

## Abstract

Solitary fibrous tumors have gene expression signatures similar to those of neuroendocrine tumors.

**Related research article** Hill CM, Indeglia A, Picone F, Murphy ME, Cipriano C, Maki RG, Gardini A. 2024. NAB2-STAT6 drives an EGR1-dependent neuroendocrine program in solitary fibrous tumors. *eLife* **13**:RP98072. doi: 10.7554/eLife.98072.

Gene fusions are common mutations that have been linked to several types of cancer, most notably chronic myelogenous leukemia, which is a result of the *BCR* gene on chromosome 22 fusing with the *ABL* gene on chromosome 9 (Nowell and Hungerford, 1960). Identifying fusion genes, and investigating the molecular behavior of the chimeric fusion proteins that arise from these mutations, may help with the diagnosis, prognosis and treatment of certain cancers.

The fusion of two genes on chromosome 12 – *NAB2* and *STAT6* – has been found in a rare form of cancer, known as a solitary fibrous tumor, which can form in almost any part of the body (Thway et al., 2016). While the initial tumor can often be removed, around 40% of them can recur or metastasize, at which point they become untreatable.

In most healthy tissues, NAB2 and STAT6 function independently. NAB2 is a transcriptional co-regulator that – being physiologically sequestered in the cytoplasm – can restrict the nuclear activity of two transcription factors involved in cell proliferation, EGR1 and EGR2 (Svaren et al., 1996), whereas STAT6 is a transcription factor that travels to the nucleus to activate gene expression (Figure 1; Hu et al., 2021). However, the molecular function of the NAB2-STAT6 fusion protein remains obscure. Now, in eLife, Alessandro Gardini and colleagues at the Wistar Institute and the University of Pennsylvania – including Connor Hill as first author – report the results of studies that help clarify the role of this fusion protein in solitary fibrous tumors (Hill et al., 2024).

**Figure 1.**
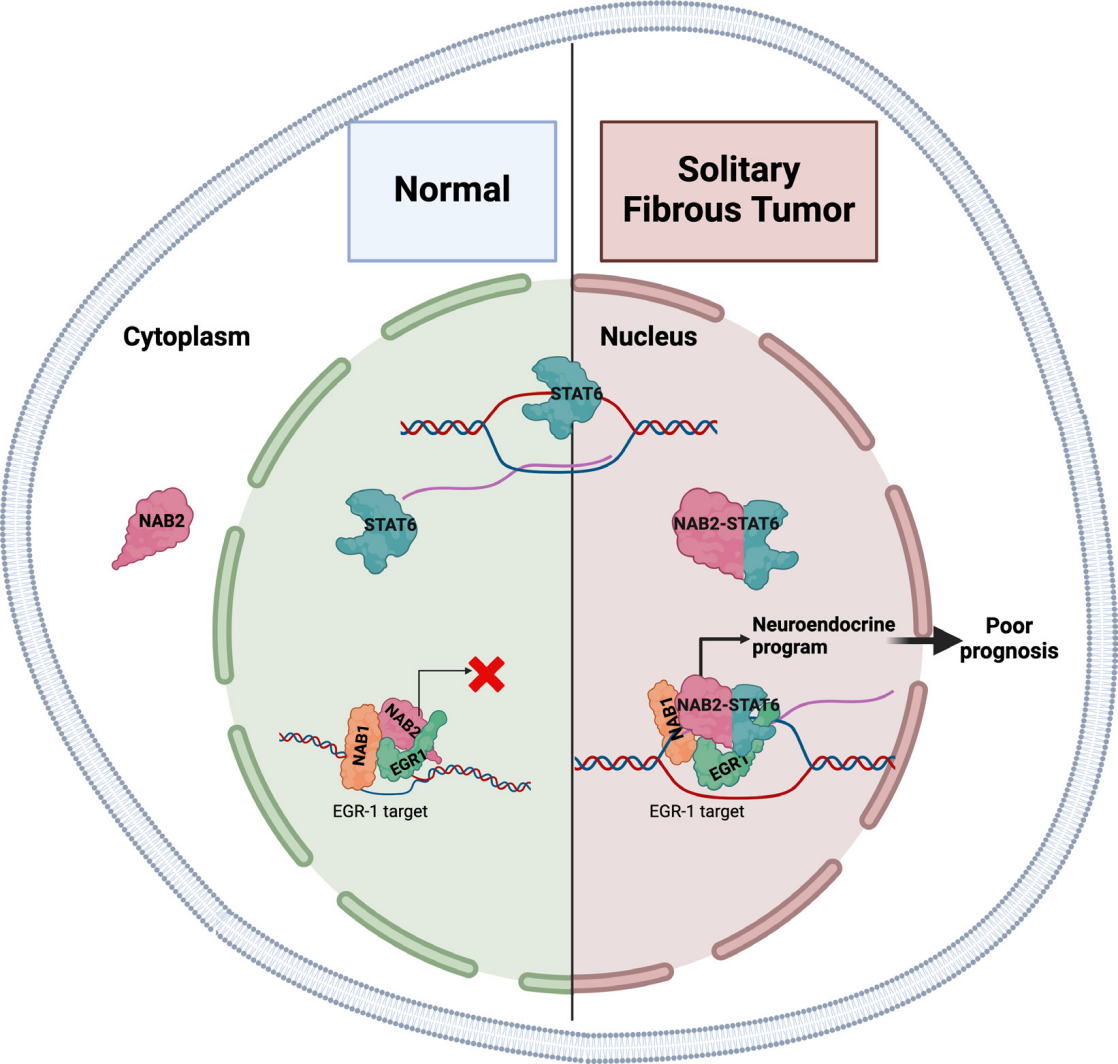
The gene expression patterns in solitary fibrous tumors are characteristic of neuronal development. In normal healthy tissues (left), the NAB2 protein is mostly located in the cytoplasm, and thus cannot co-activate EGR1 in the nucleus (red X), a transcription factor involved in cell proliferation. NAB1 is also involved in this process. STAT6 is a transcription factor that travels to the nucleus to activate gene expression. In solitary fibrous tumors (right), the NAB2-STAT6 fusion protein readily travels to the nucleus, where it binds to EGR1, and the entire complex binds to promoters and enhancers normally targeted by EGR1 (black arrow). This increases the expression of these gene targets and results in the tumors having gene expression patterns characteristic of neuronal development.

Image credit: This image was created using Biorender.

The researchers used a combination of cell-based studies, analyses of genome-wide binding patterns of specific proteins within tumor samples, and a comparative analysis of gene expression signatures. They found that compared to adjacent healthy tissue, primary solitary fibrous tumors had gene expression patterns characteristic of neuronal development, in particular, the targets of ERG1 were enriched in the tumors. Immune and cell signaling pathways were also significantly downregulated. To validate these findings, Hill et al. used cells derived from an osteosarcoma (a type of bone cancer) to create an in vitro model that expressed the NAB2-STAT6 fusion protein. This model also showed gene expression patterns characteristic of neuronal development and increased levels of NAB1, NAB2 and EGR1 bolstered by the fusion protein were also observed (Figure 1).

In both cell lines and samples from patients with cancer, the fusion protein and EGR1 bound to EGR1-targeted promoters and enhancers, increasing their accessibility and expression of the corresponding genes. The binding appeared to be mediated by the NAB2 portion, while the STAT6 portion was responsible for translocating the fusion protein to the cell nucleus.

Hill et al. then compared the gene expression signature of the solitary fibrous tumors with existing datasets from The Cancer Genome Atlas, which revealed a striking similarity between solitary fibrous tumors and neuroendocrine tumors (arising from cells that can release hormones in response to signals from the nervous system), including glioblastoma. Moreover, an analysis of registered survival rates indicated a significantly worse outcome for cancer patients with a gene signature indicative of solitary fibrous tumors.

The study of Hill et al. provides valuable insights into the dynamics of solitary fibrous tumors and their unique neural-like gene expression signature that might be relevant for other, more common cancers. Their neuroendocrine identity driven by NAB2-STAT6 fusion highlights their similarity to other neuroendocrine malignancies, such as pheochromocytoma (affecting adrenal glands) and oligodendroglioma (affecting specific glial cells in the brain), raising the question of their cell-of-origin (Hill et al., 2024; Davanzo et al., 2018; Demicco et al., 2012).

Future research on the molecular consequences of the fusion of *NAB2* and *STAT6* may help refine cancer diagnosis and inform drug development. Targeting NAB2-STAT6 or its downstream pathways could help prevent the recurrence of this cancer, or serve as a strategy for patients who are not candidates for surgery. However, more research is needed to confirm these hypotheses. Being the first of its kind, the study of Hill et al. significantly advances our molecular understanding of solitary fibrous tumors, a critical first step toward targeted precision medicine approaches.
